# Supplementary material for: Collective dynamics of dense hairy surfaces in turbulent flow
Source: Sci Rep. 2023 Mar 30;13:5184. doi: 10.1038/s41598-023-31534-7 (PMC10063604; doi:10.1038/s41598-023-31534-7)
Supplement: Supplementary file 1 — Supplementary Information. [file 41598_2023_31534_MOESM1_ESM.pdf]

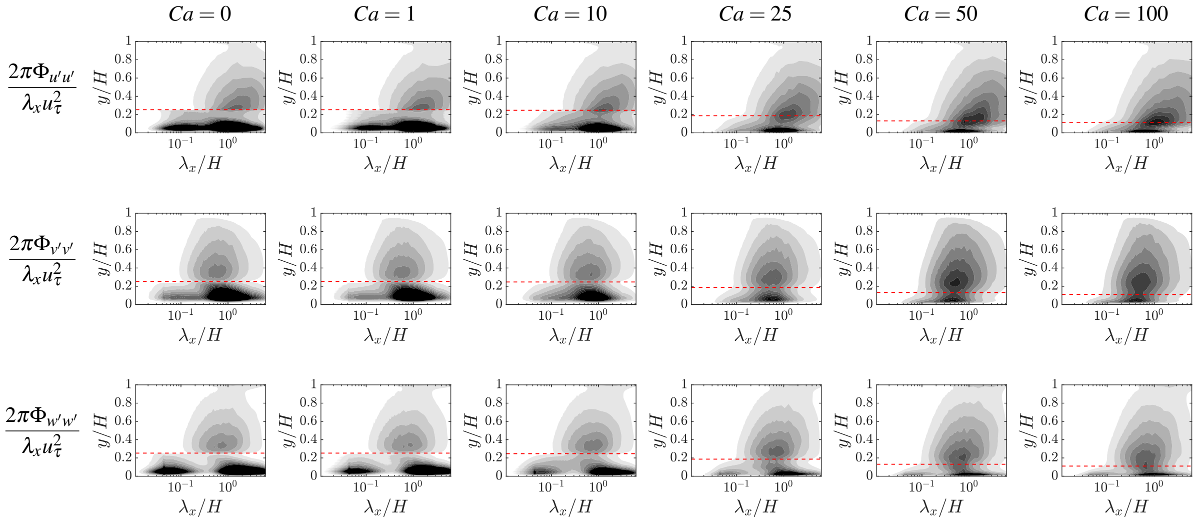

Figure S1. Magnitude of the premultiplied spectra of each fluid velocity component $2\pi\Phi_{u'u'}/(u_\tau^2\lambda_x)$, where u' the generic component of the velocity fluctuations, (top: streamwise; middle: wall-normal; bottom: spanwise) as a function of the streamwise wavelength λ_x/H and wall-normal coordinate y/H. The friction velocity is defined locally as $u_\tau = \sqrt{\tau/[\rho_f(1-y/H)]}$, where τ is the flow total shear stress, i.e. the sum of the viscous and the turbulent component.Results are shown in different columns as a function of the investigated Cauchy number (from left to right, $Ca = 0, 1, 10, 25, 50, 100$). The red horizontal dashed line indicates the averaged height of the filament tips. The grey levels range in: $[0, 0.5]$ with a 0.05 increment for the streamwise and spanwise velocity components; $[0, 0.3]$ with a 0.03 increment for the wall-normal velocity component.

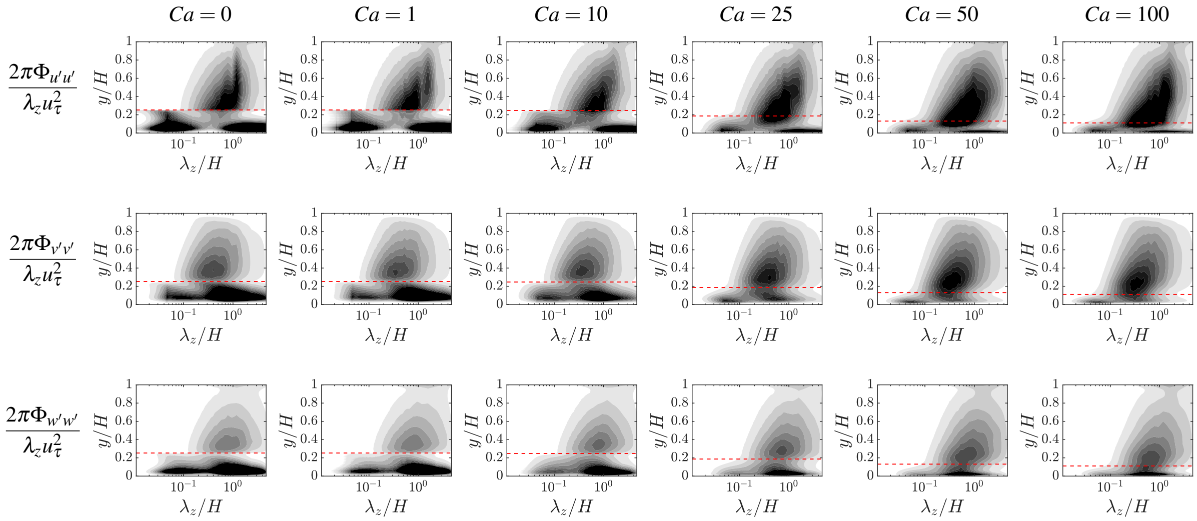

Figure S2. Magnitude of the premultiplied spectra of each fluid velocity component $2\pi\Phi_{u'u'}/(u_\tau^2\lambda_z)$, where u' the generic component of the velocity fluctuations, (top: streamwise; middle: wall-normal; bottom: spanwise) as a function of the spanwise wavelength λ_z/H and wall-normal coordinate y/H. The friction velocity is defined locally as $u_\tau = \sqrt{\tau/[\rho_f(1-y/H)]}$, where τ is the flow total shear stress, i.e. the sum of the viscous and the turbulent component.Results are shown in different columns as a function of the investigated Cauchy number (from left to right, $Ca = 0, 1, 10, 25, 50, 100$). The red horizontal dashed line indicates the averaged height of the filament tips. The grey levels range in: $[0, 0.5]$ with a 0.05 increment for the streamwise and spanwise velocity components; $[0, 0.3]$ with a 0.03 increment for the wall-normal velocity component.

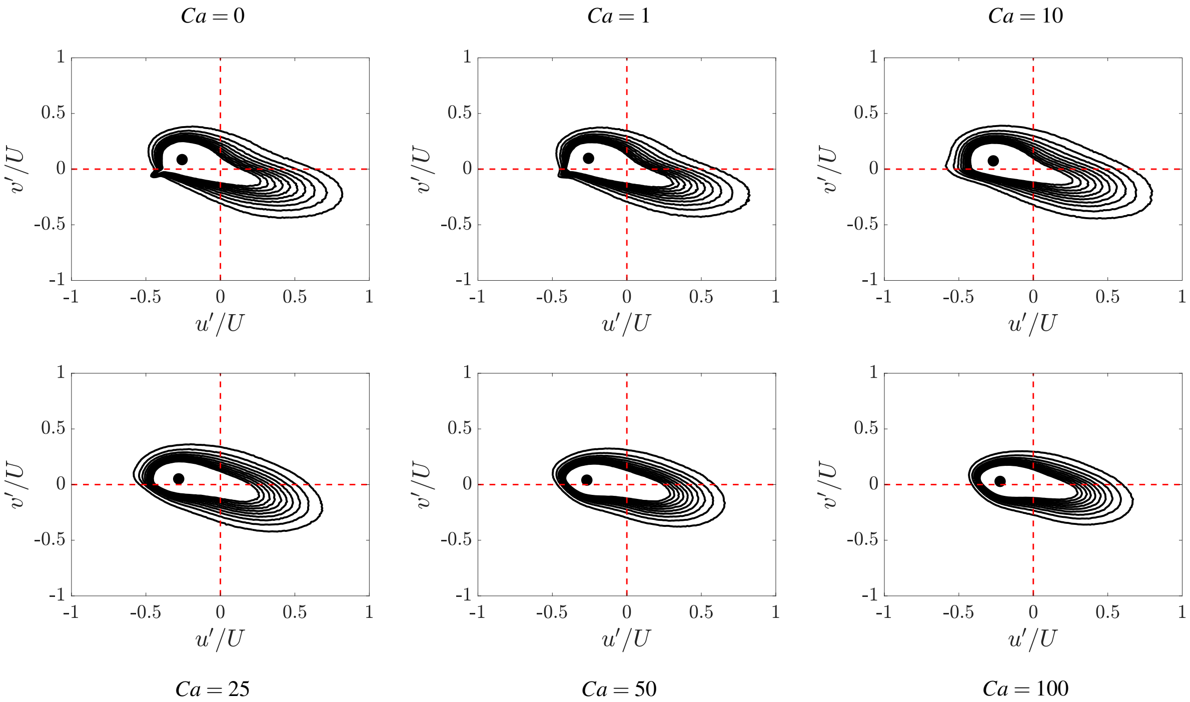

Figure S3. Contours of the joint probability density function of the fluctuations of the streamwise velocity component u'/U and of the wall-normal velocity component v'/U on a plane parallel to the wall, at $y/\bar{Y} = 1$, where $\bar{Y}$ is the average canopy height. The joint probability density function is computed for every Cauchy number. The levels of the contours range $[0 : 0.2 : 2]$, with the maximum indicated by the black circular marker (within the second quadrant). Note that the joint probability density functions are normalised such that the integral over the domain is 1. The red dashed lines represent the axes $u'/U = 0$ and $v'/U = 0$. Note that the deviation from the classic 'oval' shape close to the axis $v'/U = 0$ (with $u'/U < 0$) is caused by the wakes of the filaments.

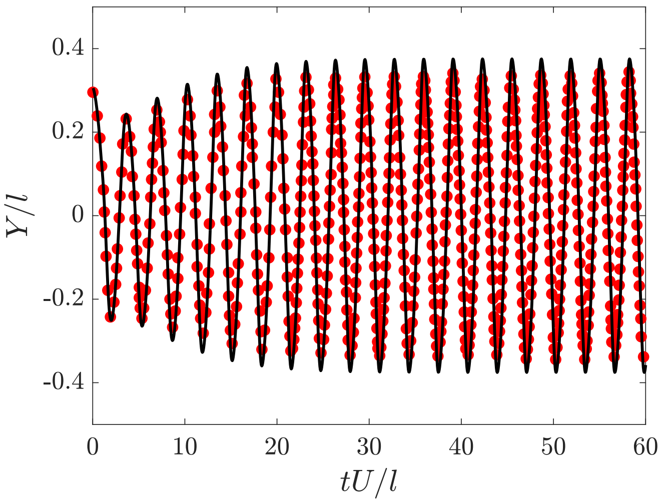

Figure S4. Validation of the employed simulation procedure for a flapping filament in uniform flow at $Re = 200$ (for more information on the problem setup, see Huang *et al.*and, in particular, figure 13 therein). Comparison of the time history of the filament's trailing point transverse position obtained with *Fujin* (black line) and that from the literature (red circles). Note that the time is normalised with the inflow velocity U and the length of the filament l, while the position of the trailing point is normalised with the length of the filament.

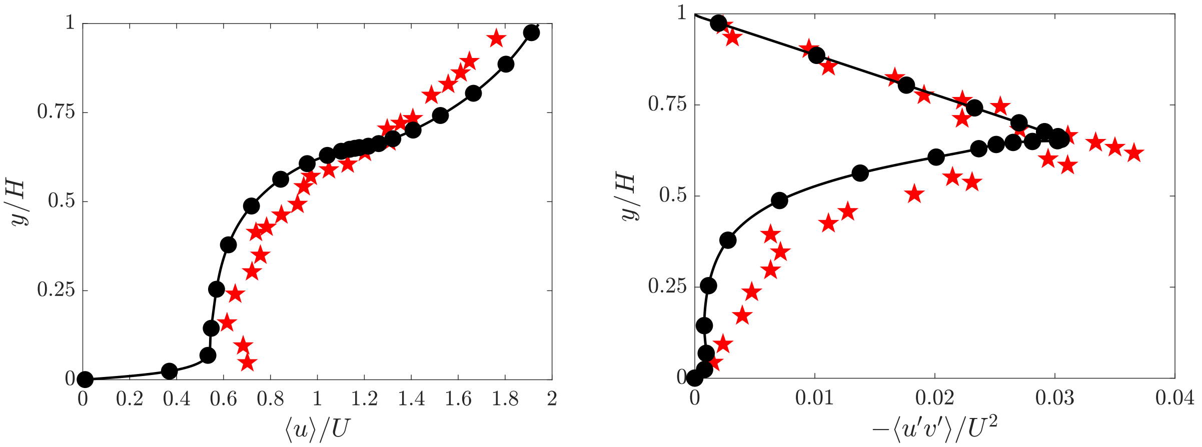

Figure S5. Validation of the employed simulation procedure for the rigid canopy case (R31) experimentally investigated by Shimizu *et al.*.Comparison between our numerical results (black circles) and the experimental measurements (red stars). Left: mean streamwise velocity profile; right: Reynolds shear stress distribution, as a function of the wall-normal location.

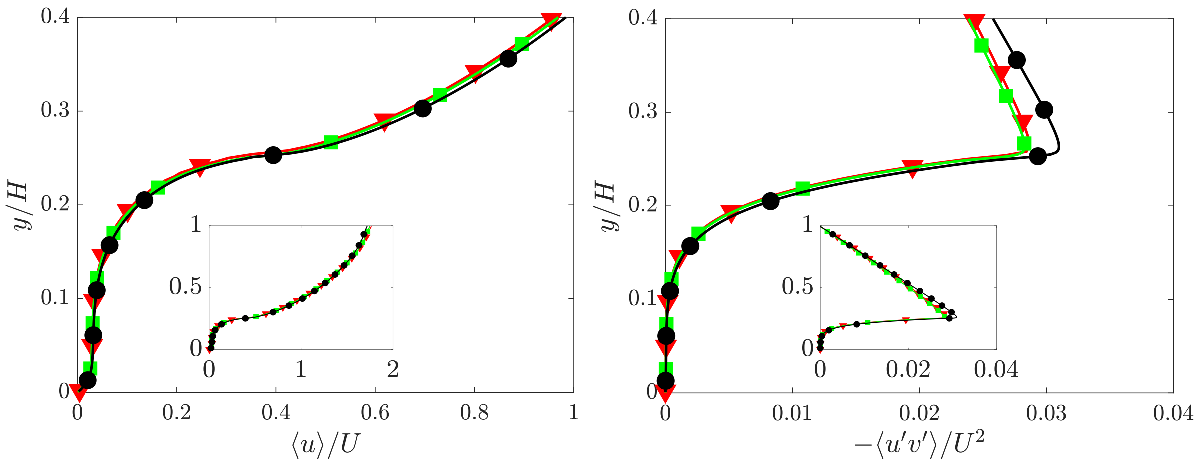

Figure S6. Convergence study over the wall-normal grid spacing for the rigid canopy case, i.e., $Ca = 0$. Comparison between the solutions obtained with halved (red triangles), baseline (black circles) and doubled (green squares) resolution. Left: mean streamwise velocity profile; right: Reynolds shear stress distribution, as a function of the wall-normal location. The main panels show a close up of the inner region (up to $y/H = 0.4$) whereas the inset panels show the full extension (up to $y/H = 1$). Note that the more evident variation for the Reynolds shear stress is arguably associated to a lack of statistical convergence rather than for the effect of the numerical resolution.
